# Surgical Site Infection Complicating Breast Cancer Surgery in Kuwait

**DOI:** 10.5402/2013/295783

**Published:** 2012-12-27

**Authors:** Abeer A. Omar, Haifaa H. Al-Mousa

**Affiliations:** ^1^Infection Control Department, Kuwait Cancer Control Center and Infection Control Directorate, Ministry of Health, Kuwait; ^2^Department of Microbiology, High Institute of Public Health, Alexandria University, Egypt; ^3^Infection Control Directorate, Ministry of Health, Kuwait

## Abstract

*Background and Objectives*. Surgical site infection (SSI) is the most common postoperative complication associated with breast cancer surgery. The present investigation aimed to determine the SSI rate after breast cancer surgeries and the causative microorganisms. *Patients and Methods*. All patients who underwent breast surgery in Kuwait Cancer Control Center as a treatment for breast cancer from January 2009–December 2010 were prospectively followed for the development of SSI. Indirect detection was used to identify SSIs through medical record to review and discussion with the treating surgeons. *Results*. The number of operations was 438. Females represented 434 (99.1%) cases while males constituted only 4 (0.9%) cases. SSIs were diagnosed after 10 operations, all for female cases. Most of the SSIs (8 cases; 80%) were detected after patients were discharged, during outpatient followup. Out of those 5/8; (62.5%) were readmitted for management of SSI. Nine patients (90%) received systemic antibiotic therapy for management of their wound infection. The SSI rate was 2.3%. The main causative organism was *Staphylococcus aureus* (*S. aureus*) which was responsible for 40% of infections. Gram negative bacteria were isolated from 40% of the cases. *Conclusion*. SSI is an important complication following breast cancer surgery. Microbiological diagnosis is an essential tool for proper management of such patients.

## 1. Introduction

Breast cancer is one of the most frequent malignancies in women worldwide [1]. In Kuwait, it ranked first among both Kuwaiti and non-Kuwaiti women. During the period from 2000 to 2008, it constituted 36.0% and 39.5% from the newly diagnosed cancer cases in Kuwaiti and non-Kuwaiti females, respectively [2]. Excision of the primary tumor (by mastectomy or breast conserving surgery) and sentinel lymph node or axillary lymph node dissection are standard procedures for the treatment of most cases [1]. Surgical site infection (SSI) is the most common postoperative complication associated with breast cancer surgery [3]. The development of SSI can lead to prolonged hospital stay with increased costs, poor cosmetic results, psychological trauma, and, occasionally, a delay in postoperative adjuvant therapies [4]. To the best of our knowledge, no published studies reported SSI rate after breast cancer surgery in Kuwait. Hence, the present investigation was aiming to determine the SSI rate after breast cancer surgeries and the causative microorganisms as a step to improve the management and the outcome of such patients.

## 2. Subjects and Methods

All patients undergoing breast surgery in Kuwait Cancer Control Center—which is a comprehensive cancer center devoted exclusively to the care of patients with cancer—as a treatment for breast cancer from January 2009 to December 2010 were prospectively followed for the development of SSI.

Indirect prospective detection of SSI was used to identify patients with SSIs [5, 6]. Patients were followed up to 30 days after surgery if there was no implant and up to one year when there was an implant placed during the operation. SSIs were identified using Centers for Disease Control and Prevention/National Healthcare Safety Network (CDC/NHSN) surveillance definition of health care-associated infections [7]. Diagnosis was based on collecting information from patients medical records including reviewing of clinical data (symptoms and signs), investigations (laboratory, histopathology, radiological, etc.), microbiological culture and sensitivity results, and medication charts in addition to discussions with the treating surgeons [5, 6]. 

Infections were identified either during the original surgical admission, at readmission to the hospital, or during outpatient follow up of the surgical wound by reviewing of medical records of surgery clinic patients [5, 6]. Causative organisms were recorded from the microbiological reports.

SSI rate during the study period was calculated using the following formula [6]:

(1) $$\begin{array}{c} \frac{\text{number}\;\text{of}\;\text{SSIs}\;\text{in}\;\text{breast}\;\text{cancer}\;\text{surgery}\times 100}{\text{number}\;\text{of}\;\text{breast}\;\text{cancer}\;\text{surgery}}. \end{array}$$

## 3. Results

The total number of operations performed for breast cancer during the study period was 438. Females represented the majority of patients, 434 (99.1%), while males constituted only 4 (0.9%) cases. Breast surgeries done were excision of the tumor either by breast conserving surgery or mastectomy accompanied with sentinel lymph node biopsy or axillary lymph node clearance. Five operations were accompanied by insertion of implant (1.1%). Surgical site infections were diagnosed after 10 operations for female cases. No prior antibiotic prophylaxis was given to any of the patients with SSI. The local policy of the center did not recommend antibiotic prophylaxis before clean surgeries. Two patients 2/10 (20%) required secondary wound closure or incision and drainage of the wound in the operating room. An-other 2/10 (20%) required bedside drainage or debridement. Most of the SSIs (8 cases, 80%) were detected after patients^,^ discharge, during outpatient follow up of the surgical wound. Out of them 5/8 (62.5%) were readmitted for management of SSI. All patients with SSI except one (90%) received systemic antibiotic therapy for management of their wound infection.

(2) $$\begin{array}{c} \text{The}\;\text{SSI}\;\text{rate}=\frac{10\times 100}{438}=2.3\;\text{per}\;100\;\text{operations}. \end{array}$$

*Staphylococcus aureus* (*S. aureus*) was the most common pathogen (isolated from 40% of all cases). Gram negative bacteria were collectively isolated from 40% of cases. Details of microbiological results were reported in Table 1. The majority (6/8 (75%)) of empirical therapy was not followed by deescalation according to culture and sensitivity results.

## 4. Discussion

Surgery has been used as part of breast cancer treatment for centuries. In Kuwait it was reported that around 75% of breast cancer cases had undergone surgery as a part of their treatment [2]. However, any surgical procedure has the potential risk of infection. The potential morbidity caused by infection can lead to serious complications. Delaying postoperative radiotherapy results in poorer control of local diseases. A significant increase in metastatic relapse and reduced survival exist when adjuvant chemotherapy is delayed [8].

We reported lower results of SSIs than reported by Degnim et al. (2.7%) [9], 4.5% by Leinung et al. [10] by Olsen et al. (4.7%) [11], and by Vilar-Compte et al. (18.9%) [12].

In our investigation, most of SSIs were diagnosed after patients' discharges. With the current trends favoring a shortened postoperative hospital stay, it becomes more likely that a significant percentage of surgical site infections will occur after these patients' discharges. Other investigators reported that an estimated 47% to 84% of SSIs occur after discharge; most of these are managed entirely in the outpatient setting [13, 14]. Minor infections are usually managed on outpatient basis while complicated wounds require readmission with the possibility of surgical interference. This is usually accompanied by delay in wound healing and consecutively delays of the start of postoperative adjuvant therapy. Most of our patients who developed SSI were readmitted for management of their complicated infections (including reoperation) adding extra cost on the healthcare settings and may worsen the patient outcome.

We believe that the rate of SSI in our study may be higher than we reported. As the postoperative length of stay is decreasing, the follow up of the patient is mainly carried out on an outpatient basis. During outpatient visits, when the SSI develops and requires no readmission, surgeons may not document the infection in the patient's records and may not request microbiological sampling of the wound. This is primarily due to fear of medical malpractice claims or negligence especially in a surgery classified as a “clean” one like breast surgery.

In the present investigation, despite the fact that* S. aureus *(MSSA and MRSA) was the primary pathogen isolated from SSIs, Gram negative bacteria were isolated in 40% of cases representing a significant finding. Other studies reported the same results [8, 15]. However, Mukhtar et al. [16] reported Gram negative bacteria as the most common isolated pathogens. These findings support the importance of the use of empirical broad-spectrum antimicrobial (not only targeting *S. aureus*) coverage (if any) until culture results become available. 

## 5. Conclusion

SSI is an important complication following breast cancer surgery. Microbiological diagnosis is an essential tool for proper management of such patients.

## Figures and Tables

**Table 1:** Distribution of pathogens causing surgical site infection complicating breast cancer surgery, January 2009–December 2010 in Kuwait Cancer Control Center, Kuwait.

Isolated bacteria	Number	%
Methicillin sensitive *Staphylococcus aureus* (MSSA)	3	30
Methicillin resistant *Staphylococcus aureus* (MRSA)	1	10
*Streptococcus pyogenes *	1	10
*Pseudomonas aeruginosa *	2	20
*Acinetobacter baumannii *	1	10
*Morganella morganii *	1	10
No pathogen isolated	1	10

Total	10	100

*Staphylococcus aureus* represented 40%.
